# Reducing Contrast-Induced Nephropathy Risk in a Murine Model: Role of Avanafil and Vardenafil in Modulating Oxidant/Antioxidant Balance

**DOI:** 10.7759/cureus.86136

**Published:** 2025-06-16

**Authors:** Charalampos Mavridis, Ioannis-Erineos Zisis, Anca Oana Docea, Ana Maria Buga, Aristidis Tsatsakis, Charalampos Mamoulakis

**Affiliations:** 1 Department of Urology, University General Hospital of Heraklion, University of Crete, Medical School, Heraklion, GRC; 2 Department of Toxicology, University of Medicine and Pharmacy of Craiova, Craiova, ROU; 3 Department of Biochemistry, University of Medicine and Pharmacy of Craiova, Craiova, ROU; 4 Department of Forensic Sciences and Toxicology, Laboratory of Toxicology, University of Crete, Medical School, Heraklion, GRC

**Keywords:** avanafil, contrast-induced nephropathy, nephroprotection, oxidative stress, vardenafil

## Abstract

Introduction: Contrast-induced nephropathy (CIN) is a major clinical problem, particularly under conditions of preexisting renal insufficiency and comorbidities. The present study evaluates the potential of phosphodiesterase type 5 inhibitors (PDE5is), avanafil (AVA), and vardenafil (VAR) to prevent CIN by modulating oxidative stress in a murine model.

Methods: Two sets of 25 male Wistar rats were allocated into five groups: control, CIN, N-acetylcysteine (NAC), VAR, and AVA. Indomethacin, L-NG-Nitro arginine methyl ester (L-NAME), and iopromide were used to induce CIN. Oxidative stress markers were evaluated, i.e., total antioxidant capacity (TAC), protein carbonyl (PROTC), thiobarbituric acid reactive substances (TBARS), glutathione (GSH), and catalase (CAT) activity.

Results: In comparison with the control group, TAC, GSH, and CAT activity were reduced, while TBARS and PROTC levels were elevated in the CIN group. Variations in treatment by VAR, AVA, and NAC induced a notable rise in TAC and blood levels of GSH while lowering TBARS in tissue.

Conclusion: The treatment groups with VAR, AVA, and NAC were noted with higher values of TAC, CAT, and GSH, while lower values of TBARS and PROTC indicated a protective effect against oxidative injury. The findings indicate that VAR and AVA effectively control the oxidant/antioxidant status, preventing oxidative stress and the incidence of CIN. Further research would be required to replicate these findings and identify the therapeutic potential of VAR and AVA in clinical conditions.

## Introduction

Contrast-induced nephropathy (CIN) is a significant complication arising from using contrast media during therapeutic and diagnostic imaging procedures such as coronary angiography and computed tomography [1]. This condition presents a significant clinical problem, especially in patients with preexisting renal insufficiency, diabetes, and other comorbidities. The development of CIN involves renal ischemia, direct toxicity to the renal tubules, and oxidative stress, which leads to an imbalance between the generation of reactive oxygen species (ROS) and the kidney's antioxidant defense systems [2].

Oxidative stress is a key factor in the initiation and progression of CIN. The overproduction of ROS can cause cellular damage, inflammation, and apoptosis within renal tissues [3]. Consequently, strategies aimed at modulating the oxidant/antioxidant balance in the kidneys can potentially reduce the risk of CIN. Phosphodiesterase type 5 inhibitors (PDE5is), such as tadalafil and sildenafil, have been demonstrated to possess antioxidant properties and enhance endothelial function [4]. In this context, avanafil (AVA) and vardenafil (VAR) have shown promising nephroprotective effects, as evidenced by measurements of creatinine, metalloproteinases (MMPs) 1 and 9, and cystatin-C (Cys-C) [5]. Furthermore, in a segmental glomerulosclerosis mouse model, VAR demonstrated nephroprotective benefits by maintaining the integrity of the glomerular vasculature, reducing podocyte loss, and improving fibrotic alterations [6]. A recent study suggested that AVA may exert an indirect renoprotective effect by increasing levels of vascular endothelial growth factor, vitamin D3, and bone morphogenetic protein 7 (BMP7) [7]. The potential nephroprotective effects of AVA and VAR have garnered substantial interest in basic research, leading to the exploration of increasingly novel pathways.

In this study, we aim to find out if AVA and VAR can decrease CIN in a murine model by modulating oxidative stress, as determined by plasma and tissue biomarkers. AVA and VAR effects are to be contrasted with the antioxidant N-acetylcysteine (NAC). Unraveling such effects has the potential to give rise to new prevention methods for clinical treatment of patients, particularly those at risk for CIN.

## Materials and methods

Reagents

All oxidative stress marker reagents, along with VAR and AVA, NAC, indomethacin, and L-NG-Nitro arginine methyl ester (L-NAME), were procured from Sigma-Aldrich (St. Louis, MO, USA), as we previously reported [4].

Animals

Twenty-five male Wistar rats (300-350 g, six months old) were sourced from the University of Medicine and Pharmacy Craiova, Romania. These rats, representing young, healthy adults, were used to eliminate aging as a variable in CIN induction. Before the experiment, rats were housed for 14 days at 21±2°C with a 12-hour light/dark cycle and had unlimited access to food and water. The experiment followed Directive 2010/63/EU standards and was approved by the Ethics Committee of the University of Medicine and Pharmacy of Craiova, Craiova, Romania (Protocol No. 32/22-05-2020). Procedures were conducted under veterinary supervision, adhering to recognized euthanasia methods.

Experimental design

After acclimatization, all the animals were divided into five equal groups (n = 5 animals per group) and treated for seven days as we previously exhibited [5]. The groups were Control: 0.5 mL corn oil daily via oral gavage; CIN: 0.5 mL corn oil daily before CIN induction; NAC: 100 mg/kg NAC in corn oil daily before CIN induction; VAR: 10 mg/kg VAR in corn oil daily before CIN induction; AVA: 50 mg/kg AVA in corn oil daily before CIN induction.

On day 6, animals were deprived of water for 12 hours. On day 7, after gavage, the CIN, CIN+VAR, CIN+AVA, and CIN+NAC groups received intraperitoneal injections of indomethacin (10 mg/kg), L-NAME (10 mg/kg), and iopromide (3 g/kg iodine) via the tail vein under light anesthesia. The control group received equivalent phosphate-buffered saline (PBS) volumes. Animals were weighed at the start and before sacrifice.

Assessment of oxidative stress indicators

We followed our established experimental design for sildenafil and tadalafil. Blood samples were collected 48 hours after CIN induction, processed to separate plasma and erythrocytes, and stored at -80°C. Plasma and tissue homogenate were analyzed for total antioxidant capacity (TAC), protein carbonyl (PROTC), and thiobarbituric reactive substances (TBARS) using established protocols with minor modifications as previously applied in our published work [4,8-10]. Erythrocyte lysates were used to assess glutathione (GSH) levels and catalase (CAT) activity following the methods of Reddy et al. and Aebi, respectively, also in line with our earlier experimental procedures [4,11,12].

Briefly, the GSH levels were determined from the erythrocyte lysate that was treated with trichloroacetic acid (TCA), phosphate buffer, and 5,5-dithiobis (2-nitrobenzene) acid (DTNB) and then incubated. Finally, the absorbance was read at 412 nm, and the GSH levels were determined compared to the DTNB extinction.

The CAT activity was determined from erythrocyte lysate mixed with phosphate buffer, incubated, and treated with hydrogen peroxide (H₂O₂). The absorbance was measured at 240 nm, and CAT activity was determined in comparison with H₂O₂ extinction.

PROTC levels were determined in plasma/tissue homogenate mixed with TCA, treated with 2,4-dinitrophenylhydrazine (DNPH), and incubated at room temperature in the dark. The supernatant was discarded, and the samples were treated with ethanol-ethyl acetate and urea and incubated. The absorbance was measured at 375 nm, and the PROTC concentration was determined in comparison with DNPH extinction.

TBARS levels were determined from plasma/tissue homogenate that was mixed with TCA and Tris-HCl, incubated, and then treated with sodium sulfate (Na₂SO₄) and thiobarbituric acid (TBA) and incubated again. Finally, the supernatant was separated, the absorbance was measured at 530 nm, and the TBARS concentration was determined in comparison with malonyl dialdehyde extinction.

TAC levels were determined in plasma/tissue homogenate mixed with sodium phosphate buffer and 2,2-diphenyl-1-picrylhydrazyl radical (DPPH*), incubated, and measured at 520 nm.

Protein concentration in plasma was measured using a Bradford assay, and hemoglobin concentration with a BC-5000 Vet auto hematology analyzer (Mindray, Mahwah, NJ, USA) [13].

Data analysis

Data were tested for normality using the Kolmogorov-Smirnov test. Depending on the distribution, comparisons were performed using one-way ANOVA or Kruskal-Wallis. ANOVA was applied to TBARS (tissue), TAC (blood), and GSH (blood), followed by Tukey's HSD post hoc test. Kruskal-Wallis was used for TBARS (blood), PROTC (blood and tissue), CAT (blood), and TAC (tissue), with Dunn’s Bonferroni-adjusted test for post hoc comparisons. A p-value of <0.05 was considered statistically significant. Analyses were conducted using IBM SPSS Statistics software version 23 for Linux (IBM Corp., Armonk, NY).

Ethical statement

The experimental protocol followed all ethical principles according to the Declaration of Helsinki. It was approved by the Ethical Committee of the University of Medicine and Pharmacy of Craiova, Craiova, Romania (Protocol No.: 32/22-05-2020).

## Results

Quantitatively, all intervention models (CIN+VAR, CIN+AVA, and CIN+NAC) exhibited differences in oxidative stress markers compared to the CIN model. Statistically significant differences between the CIN model and the control group were observed in each case. The PROCT and TBARS markers were elevated in the CIN model compared to the intervention models, while the CAT, GSH, and TAC markers were reduced.

TBARS levels

**Figure 1:**
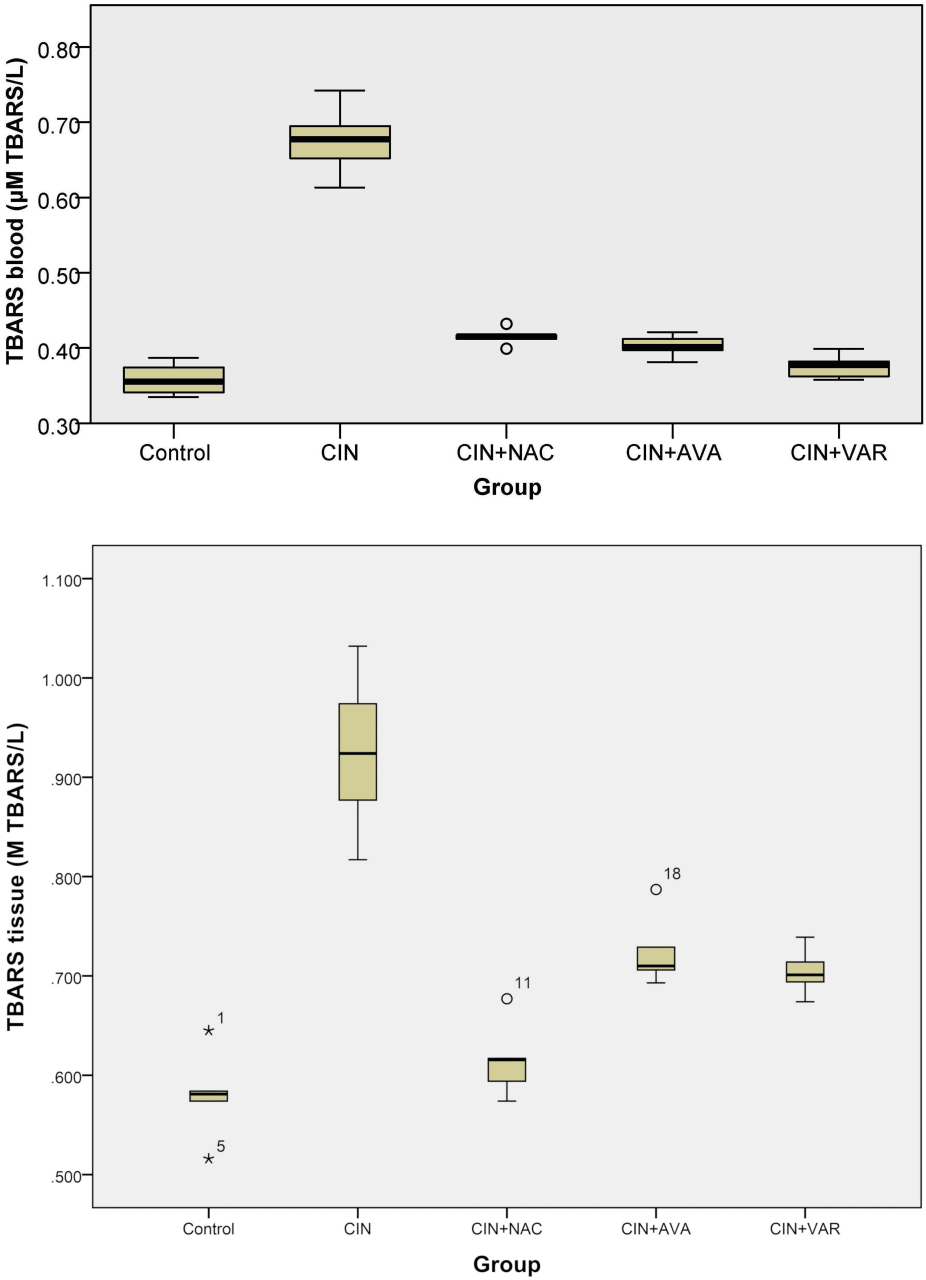
Box plots show TBARS levels in each group's blood (top) and tissue (bottom); ° indicates an outlier and * indicates an extreme outlier. TBARS: thiobarbituric acid reactive substances; CIN: contrast-induced nephropathy; NAC: N-acetylcysteine; AVA: avanafil; VAR: vardenafil

The TBARS levels in the blood of the control group did not differ significantly compared to the CIN+VAR, CIN+AVA, and CIN+NAC groups (Kruskal-Wallis, p = 1, 0.505, and 0.087, respectively). TBARS levels were elevated in the CIN group, but statistical significance was only observed compared to the control and CIN+VAR groups (p = 0.001 and 0.009, respectively) (Figure 1). In tissues, TBARS levels were significantly higher in the CIN group than in all other groups (ANOVA, p < 0.001). TBARS levels significantly decreased in the CIN+AVA, CIN+VAR, and CIN+NAC groups compared to the CIN group from (0.92 ± 0.08) to (0.73 ± 0.04), (0.7 ± 0.02), and (0.62 ± 0.04), respectively (Table 1 and Figure 1).

**Table 1:** Means and standard deviations of oxidative stress markers analyzed with one-way ANOVA CIN: contrast-induced nephropathy; AVA: avanafil; VAR: vardenafil; NAC: N-acetylcysteine; TAC: total antioxidant capacity; TBARS: thiobarbituric acid reactive substances; GSH: glutathione

Oxidative marker	Group	N	Mean	Std. deviation
TBARS tissue (μM TBARS/L)	Control	5	0.58	0.046
	CIN	5	0.92	0.083
	CIN+NAC	5	0.62	0.039
	CIN+AVA	5	0.73	0.037
	CIN+VAR	5	0.7	0.024
	Total	25		
TAC blood (mM DPPH/L)	Control	5	0.67	0.032
	CIN	5	0.36	0.032
	CIN+NAC	5	0.49	0.013
	CIN+AVA	5	0.5	0.017
	CIN+VAR	5	0.56	0.037
	Total	25		
GSH blood (μmol/g Hb)	Control	5	3.03	0.192
	CIN	5	1.45	0.135
	CIN+NAC	5	2.13	0.136
	CIN+AVA	5	2.24	0.143
	CIN+VAR	5	2.58	0.134
	Total	25		

TAC levels

**Figure 2:**
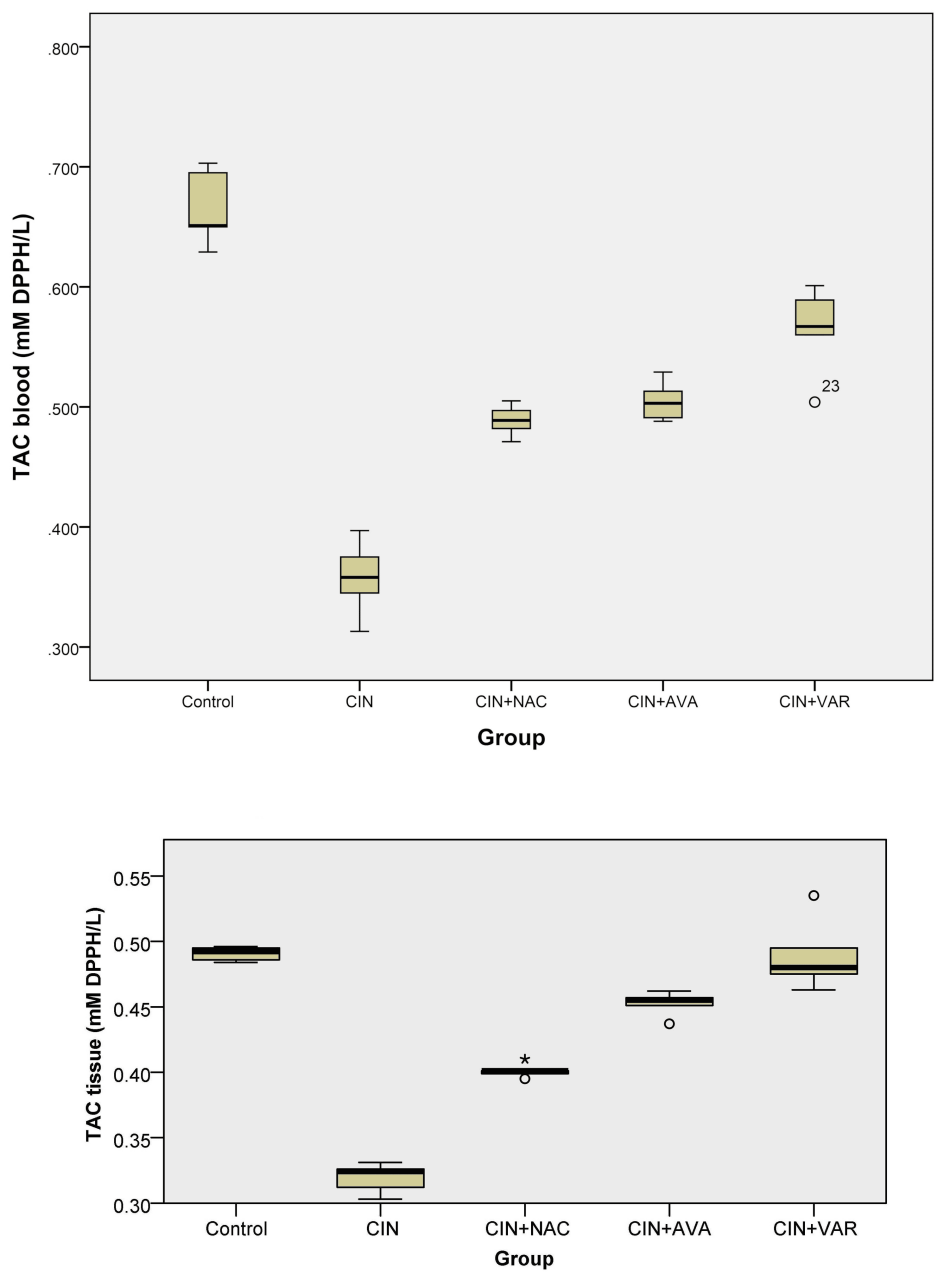
Box plots show TAC levels in each group's blood (top) and tissue (bottom); ° indicates an outlier and * indicates an extreme outlier. TAC: total antioxidant capacity; CIN: contrast-induced nephropathy; NAC: N-acetylcysteine; AVA: avanafil; VAR: vardenafil

In the blood, TAC levels were significantly lower in the CIN group than in all other groups (ANOVA, p < 0.001). TAC levels significantly increased in the CIN+AVA, CIN+VAR, and CIN+NAC groups compared to the CIN group from (0.36 ± 0.03) to (0.5 ± 0.02), (0.56 ± 0.04), and (0.49 ± 0.01), respectively (Table 1 and Figure 2). Similar results were observed in tissues, where TAC levels were significantly increased in the control, CIN+VAR, and CIN+NAC groups compared to the CIN group (Kruskal-Wallis, p = 0.001, 0.003, and 0.043, respectively) (Figure 2).

PROCT levels

**Figure 3:**
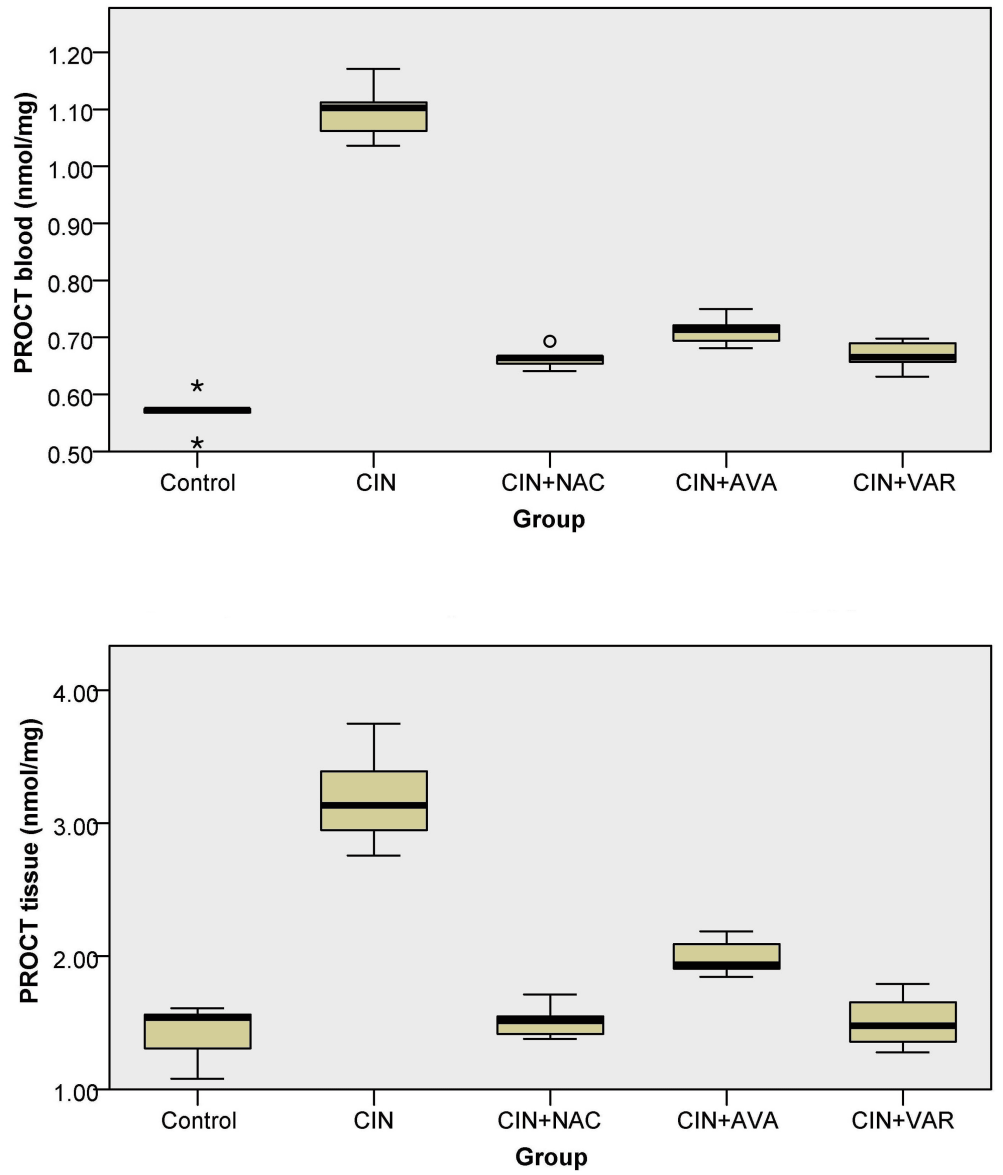
Box plots show PROCT levels in each group's blood (top) and tissue (bottom); ° indicates an outlier and * indicates an extreme outlier. PROTC: protein carbonyl; CIN: contrast-induced nephropathy; NAC: N-acetylcysteine; AVA: avanafil; VAR: vardenafil

PROCT levels in the blood were found to be significantly elevated in the CIN group compared to the control (Kruskal-Wallis, p < 0.001) and CIN+AVA groups (p = 0.023, respectively) (Figure 3). In tissues, PROCT levels were significantly increased in the CIN group compared to the control, CIN+VAR, and CIN+NAC groups (Kruskal-Wallis, p = 0.007, 0.015, and 0.02, respectively) (Figure 3).

CAT activity levels

**Figure 4:**
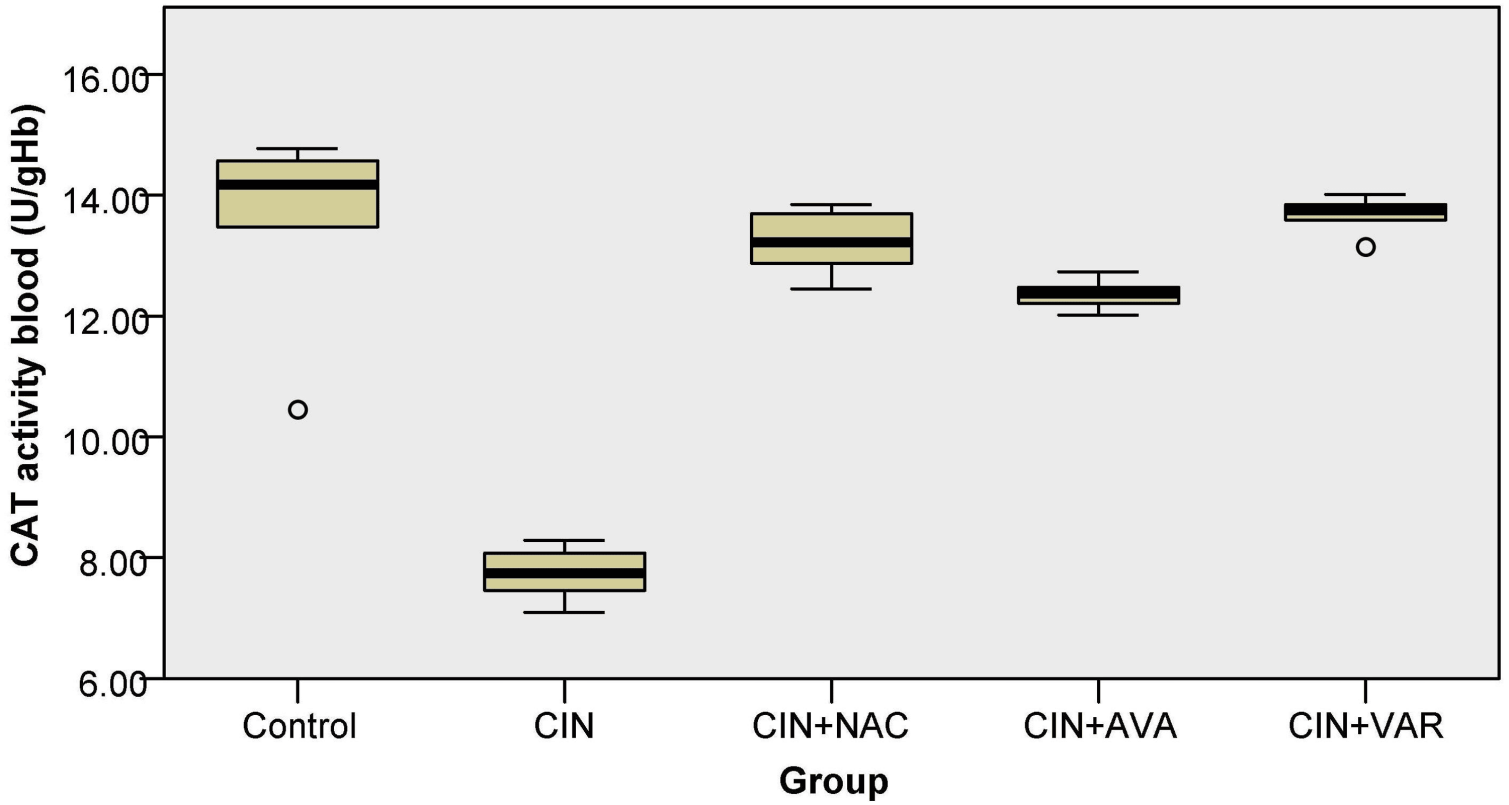
Box plots show CAT levels in each group's blood; ° indicates an outlier. CAT: catalase; CIN: contrast-induced nephropathy; NAC: N-acetylcysteine; AVA: avanafil; VAR: vardenafil

CAT activity levels significantly decreased in the CIN group compared to the control group (Kruskal-Wallis, p = 0.007). Additionally, in the CIN+VAR group, CAT activity was statistically increased compared to the CIN group (p = 0.009) (Figure 4).

Reduced GSH levels

**Figure 5:**
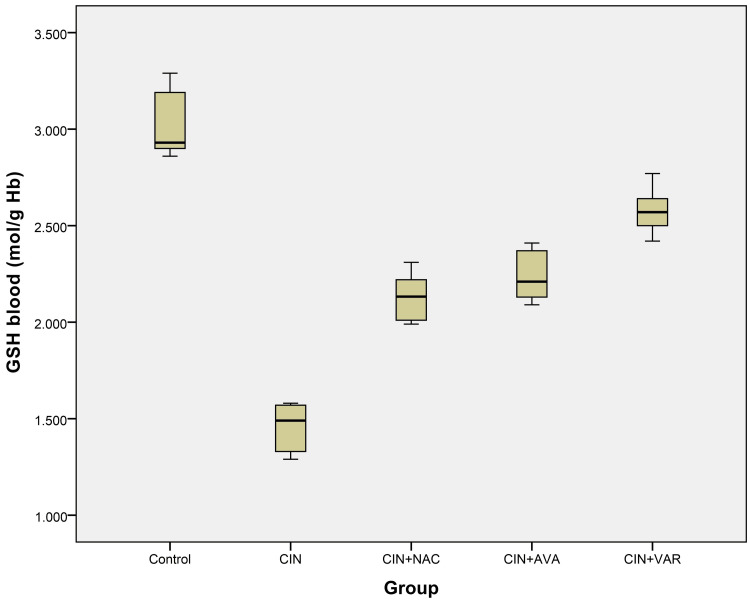
Box plots show GSH levels in each group's blood. GSH: glutathione; CIN: contrast-induced nephropathy; NAC: N-acetylcysteine; AVA: avanafil; VAR: vardenafil

GSH levels significantly decreased in the CIN group compared to the other groups (ANOVA, p < 0.001). GSH levels increased dramatically in the CIN+AVA, CIN+VAR, and CIN+NAC groups compared to the CIN group from (1.45 ± 0.13) to (2.24 ± 0.14), (2.58 ± 0.13), and (2.13 ± 0.14), respectively (Table 1 and Figure 5).

## Discussion

In the pathophysiology of CIN, oxidative stress is a key mechanism. Administration of contrast media causes renal hypoxia, a critical factor in the production of ROS in the renal parenchyma. Renal medullary hypoxia and ischemia promote excessive ROS generation, which in turn triggers inflammatory pathways (e.g., activation of the NLRP3 inflammasome) and leads to tubular cell injury [14]. Oxidative stress not only precedes but actively contributes to functional and structural renal damage in the setting of contrast-induced nephropathy, establishing a central pathophysiologic role for ROS imbalance in disease evolution [15]. The kidney mounts a compensatory antioxidant response via nuclear factor erythroid 2-related factor 2 (Nrf2), but in severe CIN, this defense can be overwhelmed; indeed, loss of Nrf2 function exacerbates contrast-induced ROS accumulation and cell death, whereas Nrf2 activation attenuates tubular injury [2]. By measuring markers that assess the severity of oxidative stress, we can evaluate the extent of renal damage. This is particularly important since renal injury from contrast media is mediated by a cascade involving oxidative stress, inflammation, and tubular damage [16]. This study assessed oxidative stress by measuring TAC, TBARS, and PROTC in blood and tissue samples and GSH and CAT in blood samples. Reduced GSH is the main non-enzymatic antioxidant involved in the control of ROS. Under normal conditions, it is balanced with its oxidized form (glutathione disulfide (GSSG)). In oxidative stress models, GSH levels decrease compared to GSSG, sometimes reaching a ratio of 1:1 [17]. CAT is an antioxidant enzyme that protects cells from damage caused by H₂O₂, a metabolic byproduct [18]. TBARS are markers of lipid peroxidation, and their levels increase during oxidative stress [19]. PROTC is a marker of protein oxidation, and its levels increase during oxidative stress. They are considered reliable indicators, as carbonyls are stable molecules [20]. TAC is a marker of the total antioxidant capacity of plasma, referring to the ability of blood plasma components to neutralize ROS [21].

In our study, blood sample levels of TAC, GSH, and CAT significantly decreased, while levels of TBARS and PROTC increased in the CIN group compared to the control group. These findings align with previous research, supporting the idea that these markers help diagnose CIN [22-25]. Recent animal data also support this diagnostic utility, as these markers correlated well with histopathological changes and novel biomarkers of injury in CIN models [26]. Regarding tissue levels of TBARS and PROCT, they were reduced in the CIN+VAR, CIN+AVA, and CIN+NAC groups compared with the only CIN group, representing a reduction in lipid peroxidation and protein oxidation. On the other hand, TAC, CAT, and GSH levels demonstrated a return toward control values, representing improved antioxidant defense mechanisms in the treatment groups.

In the therapeutic groups CIN+VAR, CIN+AVA, and CIN+NAC, TAC and GSH levels increased in blood compared to the CIN group, with statistically significant differences. The measurements of the therapeutic groups were very close to those of the control group. Additionally, while TBARS and PROTC levels significantly increased in the CIN group compared to the control group, they decreased in the therapeutic groups, approaching the initial measurements. Notably, the magnitude of improvement with AVA and VAR was comparable to that achieved with NAC, a standard antioxidant therapy for CIN, suggesting that PDE5i provides antioxidant protection on par with direct ROS scavenging. Based on these results, VAR, AVA, and NAC protect against lipid peroxidation and oxidative protein damage caused by CM administration.

Until now, VAR and AVA have not been studied in CIN models. However, previous studies have examined the nephroprotective effects of sildenafil and tadalafil against CIN. In an animal model of CIN, sildenafil reduced ROS overproduction, resulting in a positive effect against CIN [27]. In other AKI models, regardless of etiology, sildenafil and tadalafil showed nephroprotective effects after assessing the above markers [4,28]. In those studies, TAC decreased in the CIN group and increased in all therapeutic groups, while TBARS and PROTC increased in the CIN group and decreased in the treatment groups, mirroring our findings. These protective effects of PDE5is are attributed to their multimodal mechanism of action: by enhancing renal perfusion via NO-cGMP-mediated vasodilation, they improve medullary oxygenation and reduce hypoxia-induced ROS generation, and by stabilizing mitochondrial function, they mitigate oxidative stress and inflammation in renal cells [28]. VAR has been studied in a limited number of AKI animal model studies and only in a CIN model now, while AVA has not been previously studied in any AKI animal model [28]. In other kidney disease models, VAR treatment decreased the severity of glomerulosclerosis and tubulointerstitial injury, as revealed by histological analysis [6]. Besides mitigating Adriamycin-induced podocyte loss, VAR also protected endothelial cells within the glomerular capillaries and reduced collagen fiber accumulation in both the mesangial region and Bowman's capsule basement membrane [6]. Furthermore, VAR demonstrated the ability to inhibit fibroblasts' transdifferentiation to myofibroblasts induced by transforming growth factor-β [6]. Under a completely different design concerning the negative impact of dexamethasone on BMP7, vitamin D3, and vascular endothelial growth factor, zaprinast, and especially AVA, appeared to mitigate this effect [7]. BMP7 can maintain the phenotype of tubular epithelial cells, induce tissue regeneration, and reduce the inflammatory response and cell apoptosis [7]. Vitamin D3 contributed to a lesser degree of degeneration and congestion in a rat model of ischemia-reperfusion injury [29]. In agreement with previous studies, the present study provides evidence supporting the nephroprotective action of VAR by improving all examined markers and histological alterations. Also, the results are encouraging for AVA, making both specific PDE5is promising therapeutic options for CIN prevention. From a translational perspective, these agents' safety profiles and pharmacologic properties support their potential use in clinical settings. Both vardenafil and avanafil are clinically approved for other indications and generally well-tolerated; notably, avanafil’s rapid onset of action (around 15 minutes) and higher selectivity for PDE5 (relative to off-target PDE isoforms) distinguish it as an attractive option for on-demand prophylaxis, while vardenafil’s intermediate half-life (~4-5 hours) could cover the high-risk period during and after contrast exposure [30]. No adverse effects were observed in our animal model, and extensive clinical use of PDE5is has shown them to be safe, but caution is warranted in translating these findings (for example, to avoid hypotension in patients concurrently using nitrates).

This study has limitations, such as the lack of groups to investigate how VAR and AVA alone (without CIN induction) affect the evaluated parameters, which would help delineate their direct effects on redox balance. Additionally, the effectiveness of the two drugs could be further examined by varying the timing of administration. For instance, whether a single dose immediately before or after exposure or post-contrast treatment is as effective as the seven-day pretreatment used here remains to be studied. Furthermore, the small animal sample size may underpower some analyses, potentially explaining why certain differences did not reach statistical significance. Future studies should address these limitations and explore combination therapies to enhance nephroprotection. Investigating VAR and AVA as adjuncts to standard prophylactic measures (e.g., vigorous hydration, NAC, or statin pretreatment) could determine if synergistic benefits occur. Multimodal preventive strategies that target hemodynamic, oxidative, and inflammatory pathways together may offer the greatest protection against CIN. Translation into clinical practice will require carefully designed trials to verify that PDE5is can safely reduce CIN in at-risk patients, but our findings lay the groundwork for such future investigations.

## Conclusions

The present study demonstrated the close association between CIN and oxidative stress, as shown by the increase in TBARS and PROTC markers, as well as the decrease in TAC, GSH, and CAT catalase enzyme activity levels. Administration of the PDE5is, VAR, and AVA restored the antioxidant balance at the mitochondrial level and limited oxidative damage, showing activity similar to NAC. These observations indicate the nephroprotective effect of PDE5is on CIN and their potential to be developed as therapeutic agents. With good safety profiles and availability in the clinic, VAR and AVA should be considered for further larger-scale trials, as they may provide a cervical step toward strategies to prevent CIN.

## References

[1] Wu MY, Lo WC, Wu YC, Lin TC, Lin CH, Wu MS, Tu YK (2022). The incidence of contrast-induced nephropathy and the need of dialysis in patients receiving angiography: a systematic review and meta-analysis. Front Med (Lausanne).

[2] Cho E, Ko GJ (2022). The pathophysiology and the management of radiocontrast-induced nephropathy. Diagnostics (Basel).

[3] Tirichen H, Yaigoub H, Xu W, Wu C, Li R, Li Y (2021). Mitochondrial reactive oxygen species and their contribution in chronic kidney disease progression through oxidative stress. Front Physiol.

[4] Iordache AM, Docea AO, Buga AM (2020). Sildenafil and tadalafil reduce the risk of contrast-induced nephropathy by modulating the oxidant/antioxidant balance in a murine model. Food Chem Toxicol.

[5] Zisis IE, Georgiadis G, Docea AO (2022). Renoprotective effect of vardenafil and avanafil in contrast-induced nephropathy: emerging evidence from an animal model. J Pers Med.

[6] Hu SW, Wang YH, Huang JS, Yang YM, Wu CC, Cheng CW (2022). The PDE5 inhibitor, vardenafil, ameliorates progressive pathological changes in a focal segmental glomerulosclerosis mouse model. Life Sci.

[7] Huyut Z, Bakan N, Akbay Hİ, Yıldırım S, Şekeroğlu MR (2022). Zaprinast and avanafil increase the vascular endothelial growth factor, vitamin D(3), bone morphogenic proteins 4 and 7 levels in the kidney tissue of male rats applied the glucocorticoid. Arch Physiol Biochem.

[8] Janaszewska A, Bartosz G (2002). Assay of total antioxidant capacity: comparison of four methods as applied to human blood plasma. Scand J Clin Lab Invest.

[9] Patsoukis N, Zervoudakis G, Panagopoulos NT, Georgiou CD, Angelatou F, Matsokis NA (2004). Thiol redox state (TRS) and oxidative stress in the mouse hippocampus after pentylenetetrazol-induced epileptic seizure. Neurosci Lett.

[10] Keles MS, Taysi S, Sen N, Aksoy H, Akçay F (2001). Effect of corticosteroid therapy on serum and CSF malondialdehyde and antioxidant proteins in multiple sclerosis. Can J Neurol Sci.

[11] Reddy YN, Murthy SV, Krishna DR, Prabhakar MC (2004). Role of free radicals and antioxidants in tuberculosis patients. Indian J Tuberc.

[12] Aebi H (1984). Catalase in vitro. Methods Enzymol.

[13] Bradford MM (1976). A rapid and sensitive method for the quantitation of microgram quantities of protein utilizing the principle of protein-dye binding. Anal Biochem.

[14] Shen J, Wang L, Jiang N (2016). NLRP3 inflammasome mediates contrast media-induced acute kidney injury by regulating cell apoptosis. Sci Rep.

[15] Mamoulakis C, Tsarouhas K, Fragkiadoulaki I (2017). Contrast-induced nephropathy: basic concepts, pathophysiological implications and prevention strategies. Pharmacol Ther.

[16] Tsarouhas K, Tsitsimpikou C, Papantoni X (2018). Oxidative stress and kidney injury in trans-radial catheterization. Biomed Rep.

[17] Lu SC (2009). Regulation of glutathione synthesis. Mol Aspects Med.

[18] Iwase T, Tajima A, Sugimoto S (2013). A simple assay for measuring catalase activity: a visual approach. Sci Rep.

[19] Pryor WA (1991). The antioxidant nutrients and disease prevention-what do we know and what do we need to find out?. Am J Clin Nutr.

[20] Berlett BS, Stadtman ER (1997). Protein oxidation in aging, disease, and oxidative stress. J Biol Chem.

[21] Weydert CJ, Cullen JJ (2010). Measurement of superoxide dismutase, catalase and glutathione peroxidase in cultured cells and tissue. Nat Protoc.

[22] Iordache AM, Buga AM, Albulescu D (2020). Phosphodiesterase-5 inhibitors ameliorate structural kidney damage in a rat model of contrast-induced nephropathy. Food Chem Toxicol.

[23] Chu S, Hu L, Wang X (2016). Xuezhikang ameliorates contrast media-induced nephropathy in rats via suppression of oxidative stress, inflammatory responses and apoptosis. Ren Fail.

[24] Tsamouri MM, Rapti M, Kouka P (2017). Histopathological evaluation and redox assessment in blood and kidney tissues in a rabbit contrast-induced nephrotoxicity model. Food Chem Toxicol.

[25] de Souza Santos V, Peters B, Côco LZ (2019). Silymarin protects against radiocontrast-induced nephropathy in mice. Life Sci.

[26] Mamoulakis C, Fragkiadoulaki I, Karkala P (2019). Contrast-induced nephropathy in an animal model: evaluation of novel biomarkers in blood and tissue samples. Toxicol Rep.

[27] Almeida LS, Barboza JR, Freitas FP (2016). Sildenafil prevents renal dysfunction in contrast media-induced nephropathy in Wistar rats. Hum Exp Toxicol.

[28] Georgiadis G, Zisis IE, Docea AO (2020). Current concepts on the reno-protective effects of phosphodiesterase 5 inhibitors in acute kidney injury: systematic search and review. J Clin Med.

[29] Golmohammadi M-g, Ajam R, Shahbazi A, Chinifroush-Asl MM, Banaei S (2020). Protective effect of vitamin D3 and erythropoietin on renal ischemia/reperfusion-induced liver and kidney damage in rats. J Herbmed Pharmacol.

[30] ElHady AK, El-Gamil DS, Abdel-Halim M, Abadi AH (2023). Advancements in phosphodiesterase 5 inhibitors: unveiling present and future perspectives. Pharmaceuticals (Basel).

